# Connecting with the community—experiences of medical students doing homestays in rural South Africa

**DOI:** 10.3389/fmed.2025.1610160

**Published:** 2025-09-02

**Authors:** Bernhard Gaede

**Affiliations:** Department of Family Medicine Nelson R. Mandela School of Medicine, University of KwaZulu-Natal, Durban, South Africa

**Keywords:** homestays, decentralized, rural, health professions education, humanizing, community engaged education

## Abstract

**Introduction:**

This study explores the educational and personal experiences of final-year medical students at the University of KwaZulu-Natal who participated in a homestay program during a seven-week rural clinical attachment. The initiative aimed to deepen students’ understanding of the social determinants of health by immersing them in the communities they served, moving beyond traditional hospital-based training.

**Methods:**

Students were invited to participate in the homestay project, prior to their rural clinical attachment. The study draws on qualitative data from focus group discussions and interviews with participating students, exploring their experiences during the homestays. The transcripts were thematically analysed using a framework analysis approach.

**Results:**

The research highlights how living with host families in deep rural areas fostered cultural humility, empathy, and a holistic view of care. Students reported that the homestay experience humanized their clinical practice, allowing them to perceive patients as individuals embedded within complex social and cultural contexts. Many described transformative learning moments, such as navigating cultural differences and traditional healing practices, and understanding the challenges of health care in rural communities. Despite challenges—including transport issues, food adaptation, and navigating gender norms—students overwhelmingly found the experience enriching and stress-reducing, with strong relational bonds formed between them and their host families.

**Discussion:**

The study situates these findings within broader discourses on decentralized health professions education (HPE), arguing that authentic, relational learning in community settings can challenge hierarchical and urban-centric models of medical training. The homestay model facilitated a shift in educational space—from controlled clinical environments to complex, lived community contexts—enhancing students’ professional identity formation and responsiveness to rural health needs. The process of place-making seems to be key in shaping meaning making and transformational change. The homestay project demonstrated the potential of community immersion to transform medical education by fostering empathy, cultural understanding, and a deeper, authentic connection to the lived realities of patients in shaping socially accountable health professionals.

## Background

The idea of training health professionals to be responsive to the needs of their patients and communities has emerged as a central concept in health professions education (HPE) (1, 19). This has often been conceptualized to include a strong public health perspective and understanding of social determinants of health as part of many of the curricula of HPE programs. However, the theoretical positioning or didactic teaching on the ecological links between a person’s illness and the individual, contextual and cultural dimensions may be inadequate to train health professionals to be able to deeply appreciate the context of the people that they treat – and therefore to be adequately responsive to the needs of the individuals, families and communities that seek care.

In order to increase the engagement in communities, a homestay project was initiated (see below for details) that offered final year students to live in the community during a 7 week rural attachment.

## Literature

While homestays are becoming common in tourism, their application is less commonly explored in the literature in educational settings Hughes et al. (3) Where homestays for medical students has been used, the objectives for the approach has varied, such as finding a local solution to accommodation, particularly for elective students in global health or creating a setting for teaching cultural competence. Research by Crampton et al. (2) demonstrates that homestays facilitate cultural immersion and community integration during rural clinical rotations, improving students’ understanding of social determinants of health. The literature on homestays also highlights the role in enhancing clinical training experiences, particularly in rural and international settings (3).

Several studies emphasize the educational benefits of homestays (3). It was found that living with local families deepened students’ appreciation of community context in healthcare delivery. Similarly, living in the community improved cultural competency and communication skills among participants in international homestay programs (4). The depth of the engagement by the students in the community seemed to play a critical role in the positive experiences students had (3).

Cost-effectiveness represents another theme in the literature, and the significant financial advantages of homestays in tourism compared to traditional accommodation options (5), particularly relevant given rising medical education costs. Similarly, Worley et al. (6) identified homestays as a sustainable solution for housing students during distributed clinical training.

However, challenges exist. Concerns regarding professional boundaries and privacy have been identified, and noted difficulties in homestay program standardization and quality control (7).

Overall, the literature suggests that homestays potentially offer valuable educational, cultural, and financial benefits for medical students while presenting logistical and professional challenges requiring thoughtful program design and implementation.

## Context

At the University of KwaZulu-Natal (UKZN), the final year class in the medical program spends 7 weeks based in a rural hospital in the province of KwaZulu-Natal. The hospitals where students are placed are situated between 80 km and 450 km from the central medical school and are either in small rural towns or in deep-rural communities. The module is designed to cover a range of competencies beyond the clinical training, with a strong focus on generalist skills, on how to function within the health care system, and on how to engage with traditional healers and communities.

A project was initiated to offer students an opportunity to live in the community, rather than at the provided accommodation at the rural hospitals. The sites for the project were all located in deep rural areas that were part of the previous ‘homeland’ system in apartheid South Africa, i.e., part of tribal land, which generally still has much lower socio-economic status (8). The sites were distributed across the province, St Andrews Hospital in the South, Church of Scotland Hospital in Msinga, in central KZN, Mseleni Hospital and later Bethesda Hospital, in the North of the province. The sites were selected to reflect a range of contexts of the local arrangements – in Mseleni and COSH, previous international students had already stayed in the community, and so there was a context that we could learn from. At Bethesda, there was a strong linkage between the hospital and the community, but no homestays had been attempted previously. In St Andrews, the linkage between the community and the hospital was not very strong, and there was no previous experience of students living in the community. The extensive process of engagement with the communities preceded the placement of students in the selected homes that participated in this project.

Over the period of approximately 3 years, the number of participating students increased from 1 in 2016 to 21 students per block of approximately 40 students in 2017 and 2018. The allocation of students to the available space for the project was done on a first-come, first-served basis. In fact, during 2017, more than 50% of the class requested to live in the community, rather than in the hospital accommodation, but the project was not able to accommodate all of them. The main driver of the requests seemed to be the word-of-mouth spreading of information about how positive the experience was, and some students applied to do the homestay project a number of rotations in advance, long before the application process for them opened. In total, 229 students participated in the Homestay Project between 2016 and 2018.

Part of the exploration of the project included the students’ experience of living in the community, and how this influenced their learning processes in the rural block.

## Methodology

A qualitative approach was employed for the study, using focus group discussions as the main data collection tool.

### Selection of participants

The study was introduced to the students prior to the block, so that they had a chance to select to participate and ensure placement in the hospitals where the homestay project took place. All students were invited to apply, and it was shared at every meeting that the research team wanted to understand the process and experience of the students and the community in the project. As mentioned above, the students who volunteered were selected on a first-come, first-served basis, and those students who were not able to be accommodated once the spaces for the homestays were filled were not included as participants. The students were also requested to participate in the research project, but those who did not want to do so did not disqualify them from the project.

The student population at UKZN is very diverse. The selection process of students at UKZN has a strong social justice perspective and supports the selection of students from disadvantaged backgrounds by selecting 28% from schools in economically disadvantaged areas, and a large proportion of students come from rural areas. The selection of students for this project did not apply any further criteria besides what is outlined above, but the participating students reflected the student population in terms of diverse backgrounds, such as economic backgrounds, ethnic groups, or rural–urban backgrounds.

### Data collection

While the PI of the project was on the faculty of the rural block, two independent researchers, with no other relationship to the students, were involved in managing the project and conducting the interviews and focus group discussions. As part of a larger study, the homes and the students were interviewed and visited on a regular basis by one researcher. A focus group discussion with all the students participating in the homestay project was held prior to departure to the rural block and again on return, facilitated by the 2 researchers using a question guide – see Box 1. The focus group discussions and interviews with the students were conducted in English, audio-recorded and transcribed.

BOX 1 **Guide for focus group discussions**

Can you describe your experience of being involved in the homestays project?

- Why did you decide to participate in the home stays project?
- What have you liked most about the homestays project?
- What have you liked least about the homestays project?

What did you learn from the homestays project?

Do you think your participation in the homestays project has impacted on your clinical experience?

What do you think the difference is between staying in a parkhome within the hospital and staying in homes during the rural block rotation?

- Would you recommend home stays to your fellow students?
- Did you build any relationships during the homestays project? Please explain
- Did the homestays project create an opportunity for you to build relationships in the community? Please explain:
- Do you think that home stays should be extended to other sites?

What do you suggest could have been done differently in the homestays project?

### Analysis

The focus group discussions and interviews were quality checked for errors in transcription. A framework analysis approach was used in managing and analyzing the data (9). This included the steps of transcription of the data, familiarization with the transcripts of the interviews and focus group discussions, and the initial coding of the transcripts according to emerging relevant ideas (inductive process). The inductive coding was compared with the deductive codes that emerged from the literature, which had informed the focus group guides. From these steps, an analytical framework was developed that charted and indexed the data as it was analyzed against the codes. The framework was iteratively reviewed to ensure it remained comprehensive throughout the analytical process and which aided in the interpretation of the data. The matrix structure of the process offers a useful and comprehensive overview of the data and assists in the recognition of patterns, with an inductive/deductive iteration of the data, A hierarchy of themes and nodes was developed using a framework analysis in structuring the relationships and NVivo ® software was used in the analysis process.

### Ethics

The proposal for the study was approved by the Humanities Social Sciences Research Ethics Committee of UKZN (ref: HSS/0133/016), and gate-keeper permission to conduct the research was obtained from the Registrar of the University. Students were asked to give voluntary consent to participate in the study after information was given to them. Declining to participate in the research study did not influence their participation in the homestay project. The information regarding the project was initially shared via email that was sent to the whole class of students, and students were invited to an information-sharing meeting. At the meeting, questions could be asked, and additional questions would also be discussed individually, if required, and follow-up enquiries could be made.

## Results

Rich experiences were shared from the focus groups and reflections. Many of the experiences were told with humor and enthusiasm of re-telling an exciting adventure. However, a number of difficult experiences were also shared.

### Engaging in a new context

Most students felt that the homestay project was a positive experience that allowed them it understand the people they were seeing at the hospital and the community in which they worked in a more holistic manner. They came to understand the role culture and tradition played in health and medicine, and felt that the host families reduced their stress and were most welcoming. Such an environment allowed them to feel safe, and most students indicated that it shifted their preconceived ideas and that they learnt valuable life skills, which prepared them better for their role as medical doctors.

Many students expressed that the homestay experience helped them to better understand their patients that they were treating in the hospitals. This is noted in the following student’s remarks:

‘… it made me understand the patients that we have coming to the hospital I was based at, and it broadened my perspective in a way. I got to see the patient as, uh, more than just a patient with the disease that they are presenting.’ (INT 1)

The dehumanization of patients (reducing the person into a disease entity) is a strong feature of the narratives. This is juxtaposed to the humanizing experience of understanding people in their context. The medical students were often from a different cultural background to that of the community, and the homestays gave them a platform for understanding the patients’ culture:

‘I got to understand Zulu people, I am not Zulu…their culture, what they do, and also to learn about the new … so it was amazing.’ (P6, FGD 2)

Homestays allowed medical students to transition past social barriers and feel integrated into the community. One student described this by saying,

‘… we see ourselves in the community, not being alienated from our places, but being part of those places, but being able to learn medicine, being part of the community, being able to understand and help the community in advance.’ (P8, FGD4)

Medical students were able to better understand and conceptualize the cultural significance of health. This is noted in the following comment:

‘My stay in the homestay was more than hundred percent good in my perspective, in the sense that I got to integrate with the people, how they live, I also got to understand their beliefs and the way they view western medicine, not in the patient’s perspective but in the community’s perspective.’ (P2, FGD4)

The significance of traditional medicine was further supported in another student’s viewpoint:

‘…understanding how my people live like and why they do the things they do, uh if you were in there you will understand that people in rural areas still use traditional medicine and you cannot tell them otherwise, you cannot tell them do not take these medicines, they will tell you “we are African.” So, you just really do not have to shout at them when you see them at the hospital, because it’s their culture… We just have to understand and try to explain things from our perspective, western medicine, but at the same time not to judge them. (P4, FGD4)

Living among the people allowed the medical students to feel ‘more of an African with them’ (P6, FGD 4). Living in the community not only allowed the students to observe or perceive how people live, but also supported conceptualizing their identity formation in the development of a professional persona, which arises out of the authentic engagement with the members of the household, their neighbors, and the surrounding community. The experiential and relational nature offers a much deeper appreciation of and engagement in the social ecology of the households they were staying at. The same student expressed that ‘doing family medicine in the rural block you also got to learn the context, because most of the things they come with them…when you are dealing with their issues and be able to understand that when a person had an injury from playing soccer, they will start from Inyanga for ‘ukugcaba’ (form of healing by Zulu people) then come to you. They will even delay some hours before coming to you, because they live so far’. (P6, FGD 4)

### Reality of living in the community

However, students did have some challenging experiences while living with the homestay families. Largely, they were transport issues, financial issues, and conflict in the family they were staying in, and these incidents reflected the realities of issues that are common in the community. The experiences made their living environment at times an awkward one, but also reminded them of the realities of the community and challenged the romanticized conceptualization of rural livelihoods. As an example, transport was a challenge for some students particularly when on call.

‘Um the only issue that I had was the distance to the hospital. It was quite far.’ (P9, FGD 4)

Students experienced social discomfort in the homestay homes when family members were arguing with each other. They felt like intruders and felt that this conflict impacted their relationships with the family.

In a house full of boys, some medical students, who were all female students, experienced a food shortage. However, this was resolved in discussion with the host family and made everyone aware of how to live together. At times, students noted they struggled with the food that they were given. It was cooked differently from what they were used to, and they had to adapt to that. Some students felt that it was disrespectful to complain about the host family’s food, so they acknowledged that it was difficult to get used to. Similarly, one student expressed that he was raised not to challenge the cooks!

‘One of the things that was instilled within me when I was growing up is that you do not …need to fight whoever is cooking at home … you do not question, especially if it is not the one you are married to. So there was that, so I never got to engage on the food but yah I did have struggles with food. I initially adapted but at the end of the block I was really struggling. (P4, FGD 5)

Some of the female medical students mentioned that their host families expected them to cook and clean for their host families and comply with traditionally gendered social expectations, and a few female students felt that members of the host family behaved inappropriately. The reality of living in a context where social norms were significantly different from their own created a tension and discomfort for students and required them to navigate this reality.

### Feeling nurtured and cared for

Yet, most students felt that their host families embraced them and cared for them.

‘I have never had a grandmother before, so it was um the most beautiful thing to see and I actually enjoyed it, she …fed us a lot…and she cared about us.’ (P5, FGD 2)

Another student described this experience:

‘Then there was a time when I was sick and she really she took good care of me…it felt like I was really with my mum. It was nice and I am still in communication with her, she calls me, she checks up on me if I’m still ok yah.’ (P7, FGD 3)

Similarly, another student described the relationship she had with her host mom:

‘Um we had a really nice relationship and um even when I was on call I would tell her and she would um bring a meal for me at night.’ (P3, FGD 3)

### Reduced stress

Many students commented that living in the community was less stressful for them. It was like ‘…staying at home and I wasn’t worried about cooking and everything, I was just worried about my performance like waking up and go to school and come back.’ (P6, FGD 2). This allowed students to focus on their internships.

‘Your stress levels are a bit lower and you function better at the hospital… I think it affected my clinical experience positively. (P2, FGD5).

Some students felt that they did not have to worry about domestic chores (P1, FGD2), and it was described as a ‘luxury’ that came with the home stays, saying that they did.

‘…not have to worry about washing dishes and uh running out of bread. (P3, FGD5)

### Generous, social relationships with the host families

A strong sense of being welcomed and belonging was a recurring theme in the focus groups:

‘Also, you asked what our experience was, for me, it was…to realize that there is still some good in people (.) um just experiencing this family taking someone that they do not know…but they are still willing to take you in, yeah.’ (P6, FGD 2)

Likewise, another student said:

‘…I was just like their kid, so I like that a lot.’ (P7, FGD2)

The community homes was experienced like being at home away from home through the social connectedness they felt being in a family:

‘…and waking up every day with people smiling at you like you know. They made you feel like you are part of them and I had a kid to play with… and the fact that that when you coming back you are not going back to an empty room and just look at your phone. You came back and be with people who are willing to listen to you and share fun stories with you and who want to know how the hospital was, people who are actually concerned. Those are the things I enjoyed about the stay.’ (P5, FGD 5)

Having the students live in the community also gave the community members a sense of hope. The experience of living in the community as an optional part of the medical curriculum decenters the medical school as the main place for learning. The idea that the presence of the university in the community is vital underscores the potential inversion of the current hierarchy of what is considered to be the center and what is peripheral. As one student said:

‘… I honestly feel like having UKZN sending students there is beneficial to both the students and the family, … having a presence of UKZN students in the homes would bring children hope, in a sense that they would see that a person just like us has made it through all the adversities and issues that are there and they have made it into university and they are progressing…’ (INT 1)

## Discussion

The data presented above gives a detailed insight into the experiences of students becoming part of a community during the process of the homestays. The process of ‘moving out’ into the community and repeatedly making the journey between the hospital to go to the home (‘their home’) during the rural block offers an important lens through which to reflect on the idea of decentralization of HPE (10). The journey seemed to have taken place on a number of levels at the same time, and that appeared to synergistically produce a profoundly impactful experience. These shifts include.

1. *a geographic shift* (from urban to rural hospital to community), from home or residence to living in a rural village.
2. *a shift in location within the health care system* where the educational experience takes place: from central/ ‘academic’ hospitals to district hospitals and into the community.
3. and *a shift in the educational process* – from medical school and clinical training (high control, formal, experiential learning opportunities linked to clinical scenarios), to community (low level of control by educators, rich experiential learning beyond clinical context).

The experience of the students in the homestay was shaped not only by leaving the medical school campus, but also by how this challenged or resonated with their backgrounds in urban areas, privileged contexts, or for students who grew up in rural areas. For some, the homestay was a completely novel experience, while for others, it felt more like a homecoming. Shifting the educational context to the district hospital and into the community, and having an open-ended (educational) space for experiencing living in the community further shaped the process of learning (20). In all of these levels, the notion of decentralization has a connotation of giving up power and not pre-determining the process and outcomes of the experiences. Therefore, the way the students experience their time in the rural community decenters the notion of meaning-making (11) from the urban medical school campus to the deeply meaningful relationships in the rural household. The giving up of power challenges our common approaches to assessment and, as Halata & Ellaway argue, linking assessment to authentic environments may become a threat to the authenticity of the experience of the environment (12).

Authentic and deep relationships seem to be a critical component of the homestay experience. The sense of being at home while being placed in an unfamiliar environment is seen in longitudinal relationships in longitudinal integrated clerkships (LIC’s), which foreground relational learning (6). The idea of being recognized, being cared for, and being a member of the household is a profound leverage to making meaning of the local context and the wider environment of the medical placement. Even the challenging areas that students have experienced were part of the authentic environment (such as transport difficulties) that take on a deeper meaning when experienced rather than being ‘taught’ about it. Even though some of the experiences may have been challenging, in the context of the relationship, these took on a different character that allowed for engagement and resolution. In the literature on authentic learning (13) the difference between situational authenticity and cognitive authenticity is described – arguing that situations that are cognitively authentic, may lack the depth of learning if they are not also situationally authentic (14). However, as in our example, the critical role of relationships is for authentic engagements is increasingly recognized (15), the situational authenticity developed deep cognitive authenticity in how the relationships and the situation shaped the deep learning and shifting attitudes.

The active construction of a social space echoes Lefebvre’s triad of conceived (or conceptualized) space, perceived (or experienced) space, and directly lived space in which one has agency and engagement (16). Having the students immersed and engaged in the households, their lived experience dialectically “co-reproduced perceived and conceived spaces” (Pierce & Martin, p 1284). The lived experience of the authentic engagements allows the students to conceive or conceptualize a sense of place, and perceive or be witnesses to an alternative way of being. This co-creation of space with the household of the homestay stands in contrast to the perceived privileged and centralized teaching of the medical school. It legitimizes and values the periphery (11) that challenges the biomedical dominance in the curriculum.

Such analysis offers a possible way of understanding the reflection that the students were able to see the patients in the hospital as people, as neighbors, as members of the community. It reveals how dehumanized patients are when generally presented to students in the hospitals, particularly in less decentralized settings (17). The way that the students’ construct the context of the homestay seems to be an essential process in imagining the rural area and the people that live there as having great value.

The literature on transformative education (18) foregrounds the humanizing experience as part of the educational process. The account of the students’ point to the possibilities is facilitated by a number of key factors that arise from the data. Disorienting dilemmas of struggling with food, coping with conflict in the home, are part of a deeper recognition of the ‘other’. It reflects the need to experience and engage in the local context, rather than to ‘know’ of it, as a critical component for humanizing the ‘patient’ into a human being.

As students volunteered for the placement in the community, it is noteworthy that more than half of the class sought the kind of experience that the homestay project offered, including the immersion, challenges, and the profound learning process that came from this.

## Conclusion

The educational value of deeply authentic and relational experiences in communities has been demonstrated in the reflections of the students we interviewed. The homestays showed a strong potential in humanizing the professional development of the students, which has been underpinned by active placemaking and the formation of authentic relationships by the students. It opens the exploration of how we can reposition HPE into novel environments and challenges the traditional power relations and sense of control inherent in HPE.

## Data Availability

The raw data supporting the conclusions of this article will be made available by the authors, without undue reservation.
